# Proceedings of the 4^th^ BEAT-PCD Conference and 5^th^ PCD Training School

**DOI:** 10.1186/s12919-020-00191-3

**Published:** 2020-06-19

**Authors:** Laura E. Gardner, Katie L. Horton, Amelia Shoemark, Jane S. Lucas, Kim G. Nielsen, Helene Kobbernagel, Bruna Rubbo, Robert A. Hirst, Panayiotis Kouis, Nicola Ullmann, Ana Reula, Nisreen Rumman, Hannah M. Mitchison, Andreia Pinto, Charlotte Richardson, Anne Schmidt, James Thompson, René Gaupmann, Maciej Dabrowski, Pleasantine Mill, Siobhan B. Carr, Dominic P. Norris, Claudia E. Kuehni, Myrofora Goutaki, Claire Hogg

**Affiliations:** 1 Primary Ciliary Dyskinesia Centre, Royal Brompton Hospital, Sydney Street, London, UK; 2 Primary Ciliary Dyskinesia Centre, NIHR Biomedical Research Centre, University Hospital Southampton NHS Foundation Trust, Southampton, UK; 3 University of Southampton Faculty of Medicine, Academic Unit of Clinical and Experimental Medicine, Southampton, UK; 4 Division of Molecular and Clinical Medicine, University of Dundee, Dundee, UK; 5 Danish PCD & Child Centre, CF Centre Copenhagen, Paediatric Pulmonary Service, ERN Accredited, Department of Paediatrics and Adolescent Medicine, Copenhagen University Hospital, Rigshospitalet, Copenhagen, Denmark; 6 Department of Respiratory Sciences, Centre for PCD Diagnosis and Research, University of Leicester, RKCSB, Leicester, LE2 7LX UK; 7 Respiratory Physiology Laboratory, Medical School, University of Cyprus, Nicosia, Cyprus; 8 Paediatric Pulmonology and Respiratory Intermediate Care Unit, Sleep and Long-term Ventilation Unit, Department of Pediatrics, Bambino Gesù Children’s Hospital, Rome, Italy; 9 Pathology Department, University of Valencia, Valencia, Spain; 10 Molecular, Cellular and Genomic Biomedicine Group, IIS La Fe, Valencia, Spain; 11 Department of Pediatrics, Makassed Hospital, East Jerusalem, Palestine; 12 Genetics and Genomic Medicine Programme, University College London, UCL Great Ormond Street Institute of Child Health, London, UK; 13 Department of Paediatrics, Division of Paediatric Allergy, Pulmology, and Endocrinology, Medical University of Vienna, Vienna, Austria; 14 Institute of Human Genetics, Polish Academy of Sciences, Poznan, Poland; 15 MRC Human Genetics Unit, MRC Institute of Genetics & Molecular Medicine, University of Edinburgh, Western General Hospital, Edinburgh, EH4 2XU UK; 16 MRC Harwell Institute, Harwell Campus, Oxfordshire, OX11 0RD UK; 17 Institute of Social and Preventive Medicine, University of Bern, Bern, Switzerland; 18 Paediatric Respiratory Medicine, University Children’s Hospital, University of Bern, Bern, Switzerland

**Keywords:** Primary ciliary dyskinesia, Chronic respiratory disease, Multidisciplinary

## Abstract

Primary ciliary dyskinesia (PCD) is an inherited ciliopathy leading to chronic suppurative lung disease, chronic rhinosinusitis, middle ear disease, sub-fertility and *situs* abnormalities. As PCD is rare, it is important that scientists and clinicians foster international collaborations to share expertise in order to provide the best possible diagnostic and management strategies. ‘Better Experimental Approaches to Treat Primary Ciliary Dyskinesia’ (BEAT-PCD) is a multidisciplinary network funded by EU COST Action (BM1407) to coordinate innovative basic science and clinical research from across the world to drive advances in the field. The fourth and final BEAT-PCD Conference and fifth PCD Training School were held jointly in March 2019 in Poznan, Poland. The varied program of plenaries, workshops, break-out sessions, oral and poster presentations were aimed to enhance the knowledge and skills of delegates, whilst also providing a collaborative platform to exchange ideas. In this final BEAT-PCD conference we were able to build upon programmes developed throughout the lifetime of the COST Action. These proceedings report on the conference, highlighting some of the successes of the BEAT-PCD programme.

## Introduction

Primary ciliary dyskinesia (PCD) is an inherited disorder of motile cilia dysfunction associated with chronic suppurative lung disease, chronic rhino-sinusitis, middle ear disease, *situs* abnormalities and sub-fertility [1]. Current estimates suggest an incidence across Europe of 1:10,000 [2, 3], but rates as high as 1 in 400 are reported in remote and consanguineous populations [4, 5]. Clinically, impaired mucociliary clearance results in symptoms from early life [6–8]. The burden of morbidity results from mucous retention in the airways which leads to respiratory distress in the majority of new-born infants [6–8]; recurrent infections throughout life and ultimately results in bronchiectasis [9, 10]; impaired lung function [11]; and possible respiratory failure in adult life [9, 12]. Loss of cilia motility in the upper airway and eustachian tubes can lead to middle ear disease and hearing impairment, as well as problems with chronic rhino-sinusitis [9]. Subfertility in both females and males can occur due to motility defects in fallopian tube cilia and sperm flagella [13]. During embryogenesis motile cilia drive a leftward fluid flow across the embryonic node that reliably establishes *situs* [14]. Patients with PCD are consequently prone to *situs* abnormalities with *situs inversus totalis* (SIT) in 50% and *situs ambiguus* (SA) in 6–12% of patients [15, 16]. This latter group also have an increased incidence of congenital cardiac abnormalities [15].

‘Better Experimental Approaches to Treat PCD’ [17–19] is a collaborative initiative to drive advances in PCD research, that over the last 4 years has bought together a network of scientists, clinicians and patient groups in a multidisciplinary network of over 250 participants from 27 countries. We have collaborated through a series of conferences, training schools and travel bursaries to facilitate the wider dissemination of knowledge and research outputs across the field of PCD. These Proceedings report on the progress and completion of the planned deliverables of BEAT-PCD which were presented during in the 4th and final meeting held in Poznan, Poland.

Jane Lucas (UK), Chair of the BEAT-PCD network, summarised the successful completion of the objectives that were established during the inaugural conference 4 years ago [19]. Four work groups were set up to ensure completion of the tasks through collaboration, project development and consensus groups. We recognised the advantages of co-hosting the conference and training school to enhance networking between early career researchers and seniors in the field. Dr. Lucas also discussed the success of the Short-Term Scientific Mission (STSM) bursary programme, through which travel between emerging and established centres facilitated numerous successful projects and multidisciplinary collaborations.

This final conference and training school saw the contributions from multiple working groups culminate in producing consensus statements agreed across the COST action. These focussed on the standardisation of international reporting for Transmission Electron Microscopy (TEM) of cilia for diagnostic purposes [20] and accuracy of immunofluorescence for PCD diagnostics [21]. We also established clinical criteria to better recognise pulmonary exacerbations in PCD [10], performed a survey to understand variations in practice for the eradication of *Pseudomonas aeruginosa* [22] and defined physiotherapy approaches to care [23].

## Work group activities and training school – the highlights of BEAT-PCD

**Fig. 1:**
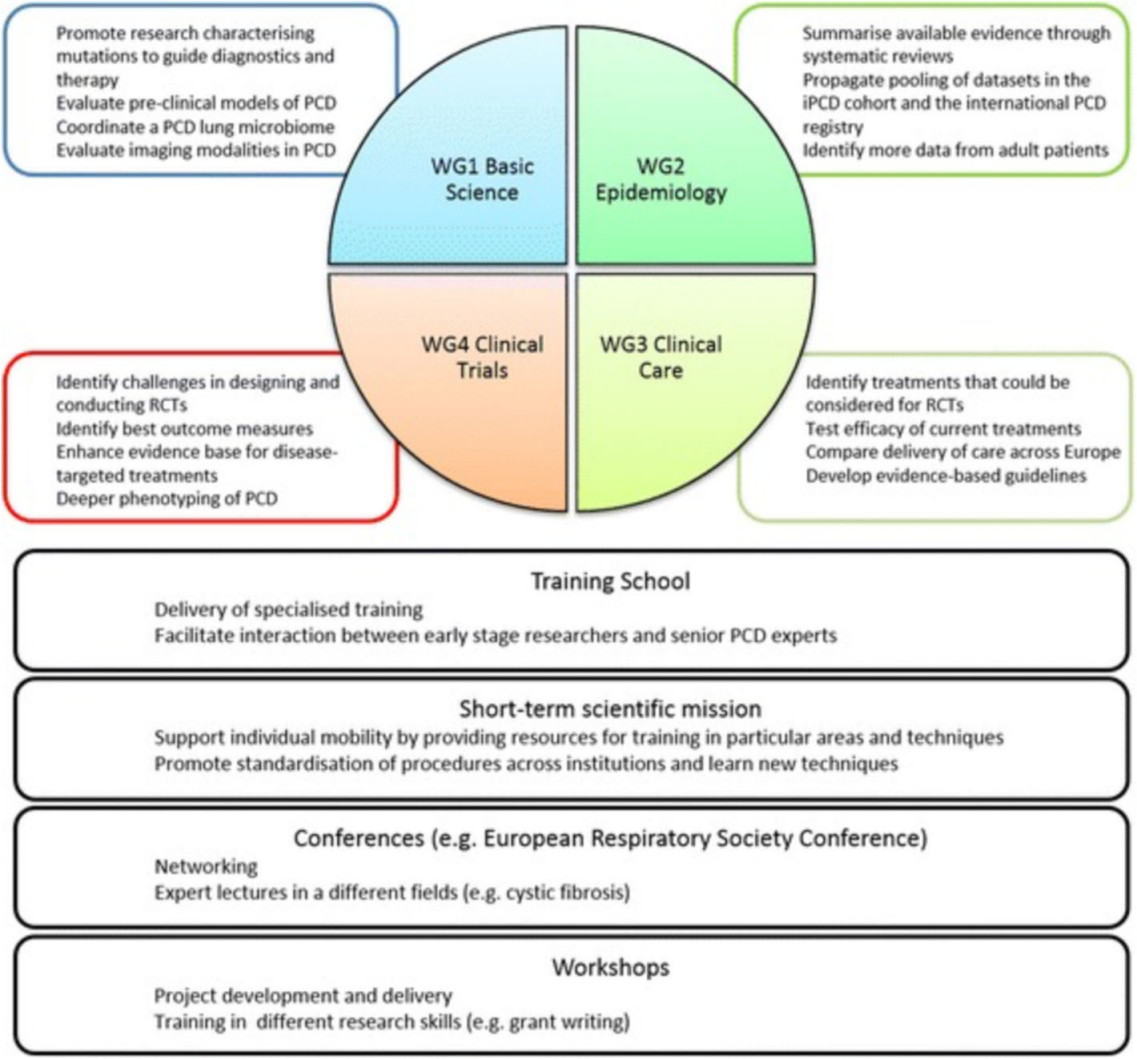
A diagrammatic representation of the aims of work groups within BEAT-PCD and COST action-associated activities. WG = work group [19].

BEAT-PCD consists of four integrated work groups: Basic Science, Epidemiology, Clinical Care and Clinical Trials (Fig. 1).

Work Group (WG1) Basic Science – lead Dominic Norris (UK): The ethos behind WG1 is ‘to get scientists to identify what is clinically important and clinicians to understand what is scientifically possible’. This group illustrates the translation of fundamental science into clinical research and ultimately, clinical practice. WG1 used various models, ranging from single celled to vertebrate systems, which have led to advances in identifying the downstream impact of specific mutations, understanding left-right patterning and the evaluation of PCD specific defects. Looking to the future, WG1 is interested in novel gene therapy approaches to rescue ciliary functionality and in exploring the PCD microbiome and its relationship with disease progression.

Work Group 2 (WG2) Epidemiology – lead Claudia Kuehni (Switzerland): A main aim of WG2 was to improve the two international collaborative PCD datasets: the international PCD (iPCD) cohort [24] and the international PCD registry [25]. The international PCD cohort (iPCD) continues to expand and now includes data from more than 3500 patients, collected from 27 centres spanning 22 countries. Several national registries and cohort studies contribute the iPCD registry [26, 27]. This has already proved to be an invaluable resource, using existing data to review diagnostic practices, growth and lung function in PCD, lobectomies, neonatal manifestations and will also explore genotype-phenotype associations [7, 28–33]. The international PCD registry is now part of the ERN-LUNG network [25]. The publication of systematic reviews and meta-analyses of published PCD data was also a success of WG2 [9, 34]. However, this revealed a clear need for the standardisation of clinical outcome measures and led to the development of a comprehensive standardised clinical form for prospective data collection (FOLLOW-PCD) by Myrofora Goutaki (Switzerland) and a large multidisciplinary team of experts [35]. FOLLOW-PCD consists of 7 modules, including a patient questionnaire on symptoms and will ensure that future studies benefit from a consistent dataset to robustly answer research questions [35]. Future areas of interest for WG2 include fertility and ear, nose and throat manifestations.

Work Group 3 (WG3) Clinical Care – lead Kim G Nielsen (Denmark): International collaboration was a key priority for WG3, this was successfully realised with more than 154 multidisciplinary clinicians and patient representatives coming together to improve PCD management. WG3 utilised surveys to consolidate knowledge and experience from experts to successfully identify topics and endpoints for meaningful clinical trials and the development of a PCD-specific evidence base. The group also focused on service delivery models, with Bruna Rubbo undertaking semi-structured interviews with experts across Europe, followed by the circulation of a survey to all delegates at the 4th BEAT-PCD conference. The PCD Physiotherapy Network also aims to improve standards of care, it now has 41 active International members and serves as an excellent platform to exchange ideas about service delivery and to support peers. The development of guidelines for PCD management through systematic review was another WG3 priority and has led to many successful outcomes. Suzanne Crowley et al. (Norway) published a survey on the treatment strategies for *Pseudomonas aeruginosa* eradication across 55 centres in 36 countries highlighting huge variation in management and the need for evidence-based guidelines [22]. An ongoing work group focussed on Infection Prevention and Control plan to produce a consensus statement in collaboration with The PCD CORE NETWORK of the ERN-Lung and patient representatives. The TEM Expert Consensus guideline involved 18 centres in 14 countries through face-to-face meetings, a Delphi consensus and exchange of samples to produce a final guideline [20].

Work Group 4 (WG4) Clinical Trials – co-led by Philipp Latzin (Switzerland) and Bruna Rubbo (UK): WG4 focussed on identifying, defining and validating outcome measures for use in longitudinal studies. Laura Behan (Ireland) and Jane Lucas (UK) developed and validated the first disease-specific quality of life questionnaires (QOL-PCD) during BESTCILIA, in collaboration with researchers from the Genetic Disorders of Mucociliary Clearance Consortium [36–40]. Ensuring QOL-PCD is both valid and robust required International collaboration; rigorous literature review [41]; expert and patient interviews with psychometric analysis to ensure the clarity and importance of each question. BESTCILIA and BEAT-PCD facilitated opportunities to undertake translation of QOL-PCD into more than 8 languages [42, 43]. Siobhan Carr (UK) and Jane Lucas (UK) led the pulmonary exacerbation consensus group. This group conducted surveys at both the 2nd and 3rd BEAT-PCD meetings followed by e-surveys to develop a definition of pulmonary exacerbation. This is characterised by the presence of three or more of the following seven items: 1) increased cough, 2) change in sputum volume and/or colour, 3) increased shortness of breath perceived by the patient or parent, 4) decision to start or change antibiotic treatment because of perceived pulmonary symptoms, 5) malaise, tiredness, fatigue or lethargy, 6) new or increased haemoptysis, and 7) temperature > 38 °C [10]. In Poznan, the group focussed on planning the validation of the consensus definition. The ‘Prospective observational multicentre study on variability of lung function in stable PCD patients’ (PROVALF-PCD) (led by Bruna Rubbo, (UK)) focussed on evaluating intra-individual variation in lung function [44]. A total of 432 patients were recruited, providing powerful data and an invaluable resource for future collaborative research and clinical care [44]. Finally, Kim G Nielsen (Denmark) shared two draft protocols for potential randomised controlled trials (RCTs) discussed in workshops held during the 3rd BEAT-PCD Conference, including the use of DNase and eradication strategies for early and chronic *Pseudomonas aeruginosa* colonisation.

## Plenaries

The success of BEAT-PCD can, in many ways, be measured by the profile of the invited speakers who were keen to come and to deliver plenary and keynote lectures. The five key-note speakers represented diverse scientific and clinical research backgrounds, and delivered their expert perspectives across a wide range of fields.

Margaret Leigh (USA) highlighted the considerable impact of international collaborations on PCD research throughout the history of the disease. By presenting a timeline, she showed the first steps, from 1904 when Siewert first described a case. She then discussed the strides taken after the first national and international PCD consortia were established, noting that prior to the formation of the Genetic Disorders of Mucociliary Clearance Consortium (GDMCC) in 2004, there were 3 case reports of PCD with heterotaxy. Afterwards, data from 337 PCD patients from 4 countries, on 3 continents, showed at least a 6% incidence of heterotaxy [45]. Subsequent studies suggest a higher incidence at 12% [16]. Gene discovery has also demonstrated the power of collaboration: the first PCD gene identified, *DNAI1*, was reported in a single centre in France [46], the third gene was described by 7 countries [47], but the *ZMYND10* gene holds the record for collaborative partnership - involving 36 institutions, 11 countries and 4 continents [48]. Professor Leigh concluded that the future of collaboration within PCD research looks bright with two multicenter randomized control trials (RCTs) (Azithromycin [49] and CLEAN-PCD [50]) due to report.

Marie Legendre (France) discussed the role of genetics in PCD diagnostics. The lack of a single ‘gold standard’ diagnostic test highlights the need to combine multiple tests to reach a definitive diagnosis [51, 52]. Whilst genetic testing can confirm a diagnosis of PCD [51], it is expensive and time consuming. A confirmed genetic diagnosis of PCD is possible in 75–80% of patients with diagnostic test results suggestive of PCD, but is as low as 22% when based entirely on clinical symptoms. Studies from the UK and France suggest that only 1.5% of young adults with bronchiectasis [53] and 5.6% of patients with recurrent airway infection [54] have a genetic diagnosis of PCD. Hence, Marie recommended that genetic testing should not yet be a first-tier test for PCD. The main challenge for the geneticist is to identify bi-allelic pathogenic PCD mutations from amongst multiple potentially rare variants of unknown significance that are regularly identified by next-generation sequencing. It is therefore essential that reporting of genetic analyses includes parental segregation, so that we can determine how often a specific variant occurs in an individual with disease within each family. Additionally, variant classification, using the universally accepted ACMG (American College of Medical Genetics and Genomics) guidelines, is important as frameshift and stop mutations are easy to classify but missense mutations can be problematic because they lead to single amino acid substitutions, with difficult to predict effects upon the structure and function of the coded protein [55, 56]. Having a single heterozygous mutation in a known PCD-causative gene is not enough to confirm the diagnosis, as the proportion of carriers for all 40 of the known PCD genes in the general population is approximately 10%. Examining genotype-phenotype associations is key to determining pathogenicity in novel gene discovery. In conclusion, at present genotyping is not an effective screening test when the suspicion of PCD is low and, if negative, genotyping does not exclude diagnosis. However, it can confirm diagnosis, particularly in difficult cases [57], but results should always be interpreted in combination with the clinical and diagnostic phenotype.

Stephanie Davis (USA) presented longitudinal data on clinical phenotypes from several North American studies [6, 58, 59]. The emergence of genotype-ciliary ultrastructural phenotype associations is now enabling clinical predictions, such as mutations in certain genes not being associated with laterality defects [15, 60]. A large cross-sectional North American study compared lung function in patients with different ultrastructural defects [59]. Participants with microtubular disarrangement, inner dynein arm or central complex defects had lower forced expiratory volume (FEV_1_) predicted compared to those with isolated outer dynein arm defects or combined outer and inner arm defects [59]. Most patients in the group with lower lung function had *CCDC39* or *CCDC40* mutations [59]. A prospective longitudinal study demonstrated that those with *CCDC39* or *CCDC40* mutations had a steeper age-associated decline in FEV_1_% predicted over a 5 year follow-up period and worse nutritional status compared to those with *DNAH5* mutations [58]. A deeper understanding of genotype-phenotype associations is essential to aid diagnosis and prognosis of PCD. Experience strongly suggests that this will happen most effectively through ongoing international collaboration.

Brian Mitchell (USA) explained the importance of planar cell polarity (PCP) in motile ciliated cells and how this provides directional information allowing coordinated beating of cilia to drive mucus in a particular direction. Loss of PCP in respiratory epithelial cells can result in PCD. He described how skin in the developing *Xenopus* embryo can be used as an experimental model to study the formation and functionality of motile cilia [61]. He explained the power of this model system to support the identification of novel human PCD candidate genes, such as *GAS2L2*. When *GAS2L2* function is depleted ciliary beat frequency (CBF) is not affected, but the directionality of beating becomes disorganised - this was observed in both the *GAS2L2* depleted *Xenopus* embryos and in the nasal epithelial cell culture of a patient with PCD carrying *GAS2L2* mutations [61]. The take home message from this talk was that PCP is important to MCC and that defects can lead to the clinical manifestations of PCD. As such, PCP of motile ciliated cells should be considered a source for potential defects, particularly in patients without clear axonemal TEM defects.

Pietro Cicuta (UK) reported on the development of video analysis for cilia stroke pattern and collective wave analysis. Differential Dynamic Microscopy allows for quantification of CBF, metachronal wavelength and cilia phase coherence in space and time. The technique is potentially advantageous because it does not require user input and can be used when it is not feasible to resolve individual objects in a given field of view. Additionally, quantitative high speed video analysis (HSVA) analysis of the beating pattern from single cilia revealed distinctive ‘fingerprints’ that can distinguish between healthy controls and samples mutated for PCD genes, such as *HYDIN* and *DNAH11*. Hence, the phenotypic parameters demonstrated with this technique not only allow for discrimination between healthy and PCD samples but also between PCD genotypes [62].

## Oral presentations

A wide variety of talks were given, through invitation and abstract submission, reflecting the cross-cutting themes that have either originated or developed through the lifetime of the BEAT-PCD COST Action. We heard about novel genes and gene-based therapies, ciliopathy syndromes and fertility. These sessions ranged from fundamental science to clinical care, including the early results from the first multicentre clinical trial [49]. These short presentations highlight how far the COST Action has come in terms of research and clinical service development across Europe through collaboration, education and spin off research projects.

### Basic sciences oral presentations

Pleasantine Mill (UK) presented data on genome editing approaches for genome therapy in PCD. Traditional gene therapy, or gene augmentation, can be hampered by the large number and size of genes implicated in the disease. CRISPR/Cas9 technology can potentially be used to target any gene, with the possibility to repair mutations via either error-free homology directed repair or alternate homology-independent methods. Unlike approaches such as antisense oligonucleotide or mRNA-based therapies, which require re-administration, genome editing therapies could be curative after a single application, if done in the airway basal stem cells. The major challenges now are to develop efficient and non-toxic in vivo delivery systems and to better control the editing events. Work was presented on developing new platforms to rapidly screen for efficacy and toxicity of both viral (lentiviral and adeno-associated viral) and non-viral delivery routes, prior to their validation in PCD mouse models.

Zuzanna Bukowy-Bieryllo (Poland) and Rob Hirst (UK) both presented on the links between PCD and ciliopathy syndromes that typically involve non-motile cilia functions. Both groups identified mutations causing respiratory motile cilia defects in patients with syndromic features. Dr. Bukowy-Bieryllo reported X-linked recessive hemizygous mutations and one de novo mutation in the *OFD1* gene, in patients with overall relatively mild features of orofaciodigital syndrome 1 (OFDS1) accompanied by PCD-like respiratory symptoms (neonatal respiratory distress, bronchiectasis, low nasal nitric oxide (nNO)) and in one case *situs inversus* (SI) [63]. Published *OFD1* mutations cause a spectrum of X-linked ciliopathies, inherited as dominant (OFDS1) or recessive conditions (Joubert syndrome (JBTS10) and Simpson-Golabi-Behmel syndrome 2 (SGBS2)) [64, 65]. Dr. Hirst also reported OFD1-like patients with combined primary cilia symptoms but PCD-typical disease features and partial syndromic features. Both groups reported similar findings of long cilia, whilst Dr Hirst presented images of cilia with bulbous tips in both OFDS1 and Sensenbrenner syndrome (CED)-like subjects. Some of the tips showed apparent accumulated microtubular structures while IFT88 protein accumulations suggested impaired intraflagellar transport.

Selective promotion of premature termination codon (PTC) readthrough can restore functional protein expression and improve symptoms in genetic diseases [66]. Maciej Dabrowski (Poland) presented data on aminoglycoside-induced translational suppression of PTC readthrough in PCD using an in vitro transcription/translation system and an in vivo transfected epithelial line [67]. In vitro results showed that the efficiency of PTC-readthrough in human embryonic kidney (HEK) cells using the dual-luciferase reporter system was low, with read-through demonstrated in 5 of the 16 mutations analysed. Readthrough efficiency was markedly lower in vivo as compared to in vitro*,* despite using higher aminoglycoside concentrations. Primary cell culture of human bronchial epithelial cells from healthy donors was used to determine the cytotoxic effects and ciliary toxicity of aminoglycosides, compared with non-aminoglycosides. This demonstrated that non-aminoglycosides (PTC-124, tylosin, amlexanox, azithromycin, TC007 and escin) showed a lack of ciliotoxicity even at high concentrations and lower cytotoxicity, in comparison to aminoglycosides. This project was funded the Polish National Science Centre 2016/23/N/NZ4/03228.

Hannah Farley (UK) presented an update of her analysis of *Pierce1* mice, which exhibit *situs* abnormalities [68]. She followed *Pierce1* gene expression with a Lac Z reporter gene, and flow in the embryonic node using fluorescent beads. This revealed that the gene is expressed in the node and that nodal cilia demonstrate a lower CBF as well as an abnormal beat pattern. TEM analysis revealed outer dynein arm (ODA) and inner dynein arm (IDA) defects in tracheal cilia of *Pierce1* mutants. Analysis of sperm flagella showed an increase in static sperm and a reduced sperm count in *Pierce1* mutant mice, but no significant TEM defects. Together these data make *Pierce1* a candidate PCD gene.

Dorota Wloga (Poland) presented data on novel proteins in cilia beat regulation using the highly ciliated *Tertrahymena* (*thermophila*) as a model. Bioinformatic screening was used to identify proteins conserved between humans and *Tertrahymena.* The *CFAP43* gene was analysed and a knockout model exhibited a 50% reduction in ciliary travel in comparison to wildtype (WT). A stiff beat was evident in the *CFAP43* knockout and a similar result was seen when a partner protein, CFAP44, was knocked out [69]. TEM analysis showed no obvious abnormalities, but cryo-electron tomography revealed missing tetherhead complexes in these mutants: these structures may have a role in regulating the activity of IDA I1α motor domains. *CCDC* proteins linking nexin-dynein regulatory complex (N-DRC) and IDA g (one of the monomeric dyneins which contribute to the molecular architecture of IDA) were also studied, with knockout models travelling 30% of the distance of WT cells. This shows that whilst defects in these signal linkers between micro-complexes cannot be identified on TEM, they appear to play a role in regulating ciliary beating.

Anu Sironen (UK) presented on PCD gene expression in spermatogenesis. The expression of known PCD genes was followed using RNASeq both during mouse spermatogenesis and in human testis [70–72]. Immunofluorescence analysis was conducted for human sperm using antibodies that detect known PCD gene products. Relatively high expression in male germ cells as well as mutations known to cause male infertility were reported for the PCD-associated dynein preassembly genes. However, there were differences in expression of axonemal structural components noted between testis, sperm and cilia. Radial spoke head 4 (*RSPH4*) expression was very low in the testis and no protein localization was identified in sperm. However, *RSPH* 1, 3 and 9 were expressed in the testis and sperm motility defects have been observed. In addition, there appear to be expression differences for axonemal dynein heavy chains in cilia versus sperm. Surprisingly, *DNAH11* and *DNAH5* exhibited very low expression in the testis, consistent with them being dispensable in sperm, while *DNAH8* expression was high in testis. Gene expression levels can be used to increase understanding of the relative importance of proteins in specific tissues. A greater understanding of PCD patient sperm development may shed a light on the role of these proteins in motile cilia.

Isabella Aprea (Germany) presented a comparative study comparing the dynein pre-assembly machinery in respiratory cilia and sperm flagella. Respiratory cilia and sperm flagella from six PCD patients with known ODA and IDA defects were analyzed using fluorescent antibodies to ciliary proteins and TEM. Sperm cells and respiratory cilia of most affected individuals presented with a loss of ODA and IDA components, including *DNAI1*, *DNAI2* and *DNALI1*. Moreover, TEM confirmed the ODA defect. In *DNAAF2* individuals, analysis of the cilia revealed absence of ODA components only in the distal axonemes, whilst sperm cells showed a loss of ODA along their entire length. The similarity of results seen in respiratory cilia and sperm cells suggest that the processes governing dynein preassembly are conserved in both cell types.

Paweł Niewiadomski (Poland) used RNAi-based loss-of-function studies coupled with immunofluorescence microscopy and semi-automated image processing to identify novel players in the ciliary transport machinery of mammalian cells. Co-immunoprecipitation and immunoblotting were used to dissect potential interactions. Results showed that a macromolecular complex not previously implicated in soluble protein transport interacts with soluble Gli transcription factors and is critical for their delivery to the primary cilium. The Gli proteins are the key effectors of hedgehog (hh) signalling and need to be shuttled into and out of cilia for hh signalling to function normally. Further, Paweł showed that Gli proteins interact with the exocyst complex and that inhibition of exocyst function blocks Gli accumulation in cilia. Paweł suggested a possible mechanism could be that the exocyst forms a scaffold for the transport of Gli proteins to the cilium base via cilium-bound vesicles.

### Clinical oral presentations

Bernard Maître (France) shared findings from a study using an automated computed tomographic (CT) scoring system to objectively quantify lung disease in adults with PCD [73]. This aimed to develop an automated algorithm that can generate a score for severity of PCD lung disease in adults. Sixty-two adult patients with PCD and available CT scans were retrospectively included. The clinical characteristics of the group included a mean FEV_1_ of 67% and almost one third of participants having had a previous lobectomy. The CTs were then reviewed using the algorithm and compared with visual CT scores (generated by trained radiologists) and spirometry results for each patient. CT-density scores showed moderate to good negative correlations with FEV_1_ and FVC with fixed thresholds, however even stronger negative correlations were observed when thresholds were adjusted for mean lung density + 1 standard deviation. In summary, generating and using an automated CT score is feasible for adult patients with PCD [73]. This score can be used in non-standardised CT examinations and correlates with spirometry parameters. However, limitations include: the algorithm misreading blood vessels as ‘lung abnormalities’ and failing to identify air trapping as abnormal. Maître and colleagues are hoping to use the score as an objective outcome parameter in a clinical and research context.

Bruna Rubbo (UK) presented on the accuracy of high-speed video analysis (HSVA) to diagnose PCD [74, 75]. Three scientists analysed 120 movies of cilia, having been blinded to the diagnosis and other test results. A random sampling of patients from multi-disciplinary team (MDT) outcomes were selected (50% PCD, 30% highly unlikely, 20% inconclusive). Results for PCD diagnosis were stratified by comparing outputs from scientists to the standards described in the ERS diagnostic guidelines [76] (100% sensitivity and 96.2% specificity) and MDT outputs (96.7% sensitivity and 91.1% specificity). All three scientists agreed with each other for the PCD positive cases, whilst less agreement was seen in cases with inconclusive findings according to the MDT and the ERS diagnostic guidelines. Re-analyses of a random sample of 20 videos a year after the study was conducted revealed good intra-observer agreement. Following this study, the archived video library can now be accessed within the UK training centres for HSVA training purposes.

Nisreen Rumman (Palestine) shared her experience of setting up a PCD service in Palestine. Nisreen received support from the COST Action, ERS and Circassia to visit Southampton and University College London in the United Kingdom. At present, clinic resources are limited and only patients with a high suspicion of PCD (high PICADAR score [8] or low nNO) receive diagnostic testing – this is achieved in collaboration with TEM services in Southampton and genetic analyses at University College London Great Ormond Street Institute of Child Health. Of 142 samples sent for TEM, 38 were abnormal, and in the majority, a genetic mutation was identified. Six patients with normal TEM showed genetic abnormalities (five with *DNAH11* and one with *HYDIN* mutations). The majority had consanguineous parents (88%); two thirds had neonatal respiratory distress syndrome (NRDS). *Situs inversus* was present in 54, 11% have congenital heart disease, 2% had undergone previous lobectomy. Screening relatives of these index patients allowed for identification of another 25 individuals with PCD. This highlights that a functioning diagnostic service can be set up in less than 4 years through International collaboration. There are ongoing plans to establish HSVA, TEM, IF and genetic testing locally in Palestine in the near future.

Ewa Zietkiewicz (Poland) reported epidemiological data on the genetic basis of PCD in Poland, including the frequency of genetic profiles and detection efficiency in the Polish population. *Situs* abnormalities were present in 40% of cases identified from the PCD reference centre at Rabka but less than 25% from other Polish centres. All the patients included in this study underwent genetic screening for PCD mutations, regardless of supporting diagnostic data, including a panel of 21 PCD genes and whole exome sequencing in 120 patients. Unsurprisingly, access to diagnostic tests is easier in the reference centre (90% of diagnoses from Rabka were supported by at least one of nasal nitric oxide (NNO), TEM or HSVA), whilst in the other health centres these investigations were performed in only one third of cases. One hundred thirty-four pathogenic mutations have been detected in the population to date. One hundred and two of these mutations were specific to either individuals or families (and therefore individual mutations occurred at low frequency). However, of the recurring mutations, there appeared to be a potentially ‘Polish-specific’ profile. The most frequent pathogenic variant was *SPAG1* (exon 16), which has previously been described in patients of Northern European ancestry [77]. There was also a relatively low contribution from *DNAH5* sites when compared with other populations [78]. Mutations were identified in 65% of the cohort but Ewa speculated that reduced access to diagnostic PCD tests in the local health centres may have led to over-diagnosis in some cases, and therefore a reduction in gene detection rates.

Myrofora Goutaki (Switzerland) presented data on the prevalence of neonatal manifestations in PCD from 13 datasets of the iPCD multinational cohort [7, 24], with almost complete datasets for the neonatal period. One thousand one hundred and seventy-five patients had information on NRDS and 1053 had information on any neonatal manifestation. The prevalence of NRDS was 40% and of any neonatal manifestation (including rhinitis, cough, oxygen or ventilation requirement, hydrocephalus or admission to neonatal intensive care) was 66%. After adjusting for sex and laterality, mean age of diagnosis was lower in patients who reported neonatal symptoms (12 versus 14 years, *p* < 0.001). Increased awareness and close collaboration with neonatologists is needed to diagnose PCD earlier in these patients.

### ERN-lung PCD core and COST action 2

The EU funded European Reference Network (ERN) project is a patient-centric, international scientific and clinical network committed to the development of knowledge bases for rare and complex disease. There are currently 23 ERNs in place and Heymut Omran (Germany) delivered a presentation on the benefits of being part of the PCD-CORE in ERN-Lung. Health care centres must meet a set of minimal requirements to apply for full or supporting membership in the PCD CORE, including a minimum number or patients served by the centre and access to central diagnostic tests.

As the BEAT-PCD Cost Action comes to a close, we looked to the future with discussions of the next steps for this thriving research community. We are dedicated to continuing the exceptional work of BEAT-PCD with an application for a collaborative Cost Action 2 called Precision Medicine for Motile Ciliopathies (PreMedCILIA). This will aim to center around the translation of motile ciliopathy research into individualised care and therapeutic approaches for our patients, with the intention of attracting input from the pharmaceutical industry.

### Revisiting BESTCILIA: the azithromycin study

BESTCILIA was a research programme funded by the European Commission 7th Framework Programme which preceded BEAT-PCD. The initial results of the highly anticipated phase III RCT of azithromycin maintenance therapy were presented by Helene Kobbernagel and Kim G Nielsen. This double-blind, parallel group study is the first multinational pharmacotherapy RCT in PCD and it aimed to examine the efficacy and safety of azithromycin maintenance therapy for 6 months, compared to placebo [49]. Despite being slightly underpowered for the primary outcome, the results were promising regarding exacerbations. At the time of the conference further analyses were ongoing and publication was pending. The study, together with the previous trial on hypertonic saline, again highlights that recruitment is challenging in this rare disease and therefore a multinational collaborative approach is required [79, 80].

## PCD training school highlights

Claire Hogg (UK), PCD Training School Lead, discussed the evolution of the training school which first met in Bern, Switzerland in 2015 with approximately 30 delegates. Since then it has merged with the BEAT-PCD conference and grown with a powerful momentum. Over the years it has facilitated the career development of early career researchers through an important number of short-term scientific missions (STSMs) [28]. The bursary scheme which facilitates travel to-and-from specialist PCD centres has increased cross-site collaborations, advanced diagnostic applications and resulted in the creation of new diagnostic centres across the world. Some STSMs were given to senior scientists to transfer knowledge and experience but the majority were given to early career researchers who travelled to learn new techniques for a wide range of applications (diagnostic, clinical and research). Twenty-nine STSMs have taken place throughout BEAT-PCD, 11 of which occurred in year 4, including destinations such as Tubingen, Lisbon, Palestine and the UK. Many of these were presented in the BEAT-PCD poster session detailed in Table 1. In summary, the training school, its meetings and STSMs have contributed towards the training of a new generation of scientists from multiple fields involved in PCD research who will ultimately be responsible to take this consortium forward.

**Table 1:** A summary of the posters presented at the 4th BEAT-PCD Conference, including poster titles and authors

Poster Title	Authors ***(Country of first author)***
A review of the care of children and young people with Primary Ciliary Dyskinesia (PCD) in a specialist PCD service in England	Wilkins H, Harris A, Baynton L, Bright V, O’Callaghan C, Carr S, Chetcuti P, Copeland F, Driessens C, Friend A, Hogg C, Kang R, Kenia P, Kewell C, Lucas J, Marsh G, Moya E, Narayanan M, Packham S, Parsons A, Rubbo B, Truscott A, Waller K, Walker W (*United Kingdom)*
The International PCD Physio Network	Schofield LM, Bright V, Kang R, Marsh G, Wilkins H *(United Kingdom)*
Prescription patterns of inhaled corticosteroids in children with primary ciliary dyskinesia in Austria and the UK: a 2-center comparison	Gaupmann R, Cobb K, Richardson C, Marsh G, Lee K, Jamalzedeh A, Bush A, Hogg C, Carr S, Dehlink E *(Austria)*
Doctors’ treatment recommendations for Swiss patients with Primary Ciliary Dyskinesia: a retrospective cohort study	Wisse A, Goutaki M, Halbeisen FS, Barben J, Casaulta C, Clarenback C, Jung A, Latzin P, Lazor R, Lin D, Lurà M, Rochat I, Kuehni CE *(Switzerland)*
The Swiss Primary Ciliary Dyskinesia Registry (CH-PCD): an update	Goutaki M, Halbeisen FS, Wisse A, Barben J, Casaulta C, Clarenback C, Jung A, Latzin P, Lazor R, Lin D, Lurà M, Rochat I, Tschanz SA, Maurer E, Kuehni CE *(Switzerland)*
Clinical outcome measures for use in primary ciliary dyskinesia: a scoping systematic review	Rubbo B, Gahleitner F, Jackson CL, Goutaki M, Halbeisen F, Hueppe JF, Behan L, Thouvenin G, Kuehni C, Latzin P, Lucas J *(United Kingdom)*
Validation of paediatric health-related quality of life instruments for primary ciliary dyskinesia	Behan L, Leigh M, Dell S, Quittner A, Lucas J *(United Kingdom)*
Development and psychometric validation of the Greek version of the adult QOL-PCD questionnaire: preliminary results	Ioannou P, Kouis P, Middleton N, Kakkoura M, Kaliva M, Toliopoulou A, Behan L, Lucas J, Yiallouros P *(Cyprus)*
Early Lung Disease: Multiple Breath Washout to assess Lung Clearance Index in preschool and school children with PCD in comparison to CF and healthy controls	Roehmel JF, Doerfler F, Staab D, Mall M *(Germany)*
Increased resting energy expenditure in children with Primary Ciliary Dyskinesia	Ullmann N, Diamanti A, Lo Scalzo L, Cremisini Carosi F, Pizziconi C, Cutrera R *(Italy)*
Lung Structure and Function in PCD – how to best monitor disease progression?	Rodrigues T, Lopes C, Bárbara C *(Portugal)*
Developmental and behavioural problems in preschool-aged primary ciliary dyskinesia patients	Zengin Akkus P, Gharibzadeh Hizal M, Ilter Bahadur E, Ozmert E, Eryilmaz Polat S, Ozdemir G, Karahan S, Yalcin E, Dogru Ersöz D, Kiper N, Ozcelik U *(Turkey)*
3D printed models as an educational tool depicting ciliary changes in Primary Ciliary Dyskinesia (PCD)	Thompson J, Jackson C, Doherty R, Lucas J *(United Kingdom)*
STSM: Diagnosis of Primary Ciliary Dyskinesia using advanced techniques: High Resolution Tomography	Pinto A, Daudvohra F, Shoemark A, Rasteiro M, Lopes S *(Portugal)*
Whole exome sequencing reveals novel mutations in Turkish siblings with PCD	Arik Sever E, Karadag B, Sezerman OU *(Turkey)*
Increasing the diagnostic yield of whole exome sequencing in primary ciliary dyskinesia through targeted copy number analysis	Bell BST, Pengelly RJ, Collins AR *(United Kingdom)*
Genetic characterisation of Spanish patients with Primary Ciliary Dyskinesia	Camats-Tarruella N, Fernández-Cancio M, Baz-Redón N, Rovira-Amigo S, Garrido-Pontnou M, Antolin M, Ruela A, Armengot-Carceller M, Escribano A, Dasi F, Moreno-Galdó *(Spain)*
Do mutations in LRRC56 cause human motile ciliopathies?	Hirst R, Watson C, Rutman A, Williams G, Chetciti P, Sheridan E, O’Callaghan C *(United Kingdom)*
Characterisation of a new CCDC40 zebrafish mutant line generated by CRISPR-Cas9	Rasteiro M, Pinto A, Lopes S *(Portugal)*
Preliminary results of high-speed video-microscopy and immunofluorescence analysis in a Spanish cohort of patients with primary ciliary dyskinesia	Baz- Redón N, Rovira-Amigo S, Camats-Tarruella N, Fernández-Cancio M, Garrido-Pontnou M, Antolin M, Ruela A, Escribano A, Dasi F, Armengot-Carveller M, Moreno-Galdó A *(Spain)*
iPSCs-derived airway epithelium for primary ciliary dyskinesia modeling and investigation of personalized medicine	Mianné J, Ahmed E, Bourguignon C, Fieldes M, Vachier I, Bourdin A, Assou S, De Vos J (France)
Oxidative profile characterisation in nasal epithelial cells of PCD patients. Development of a new diagnostic algorithm	Ruela A, Castillo S, Herrera G, Escribano A, Armengot M, Dasi F *(Spain)*
STSM in Lisbon: Analysis of the cilia-related phenotypes in CFAP300-knockdown zebrafish	Rabiasz A, Rasteiro M, Lopes S, Witt M, Zietkiewicz E *(Poland)*
STSM in Tübingen: Training in small-scale quantitative proteomics of ciliary proteins	Bukowy-Bieryllo Z, Beyer T, Boldt K, Ueffing M, Witt M, Zietkiewicz E *(Poland)*

Poster titles presented by authors (country of first author) at the 4th BEAT-PCD Conference

### Training school workshops

#### Using the standardised PCD clinical follow-up form (FOLLOW-PCD) for and QOL-PCD in clinical care and research

Laura Behan (UK) and Myrofora Goutaki (Switzerland) led this workshop with the aim of familiarising the delegates with these two instruments of data collection. They revisited the development, validation and translation of these tools into clinical practice and reviewed available versions that can be accessed for patients. Laura Behan demonstrated how to deliver and score the QOL-PCD questionnaire during an interactive session [36–38, 40]. Myrofora Goutaki showed how to navigate FOLLOW-PCD with hands-on experience of entering patient data and extracting their datasets [35]. This workshop gave an insight into how these tools can be used in research and clinical practice.

#### Nasal NO in infants

Eric Haarman (Netherlands) and June Marthin (Denmark) led this session which highlighted the importance of early PCD diagnosis and the diagnostic value of nNO in infants. Use of nNO as a diagnostic test is well established in school age children and adults [52, 81–85]. It is important to understand the factors which influence the interpretation of nNO in infancy. Nasal NO is initially extremely low in healthy infants but increases considerably in the first few months of life [86–88]. It has also been shown that transient reductions in nNO (even below the expected cut-offs for PCD) can occur during to respiratory tract infections in infants [86]. It was therefore suggested that age-standardised nNO normative values are urgently needed for healthy and PCD infants, with regulation of measuring techniques for this group. Particular focus was given to the findings of the recent proof of concept study which demonstrated the feasibility of different methods to measure nNO in infants [89]. Three suggested methods include: the conventional method of tidal breathing for 30 s, measuring 3 peaks; tidal breathing for 4 s, measuring 1 peak and vacuum sampling using the pinch manoeuvre over 1–2 s. The workshop included an interactive session demonstrating the NIOX VERO machine and a presentation of practical techniques to improve tolerance of the technique in the infant group. The workshop generated discussions regarding a possible multicentre study to determine the discriminative ability of nNO in infants.

#### Systematically analysing genotype-phenotype association in mouse: mining international mouse phenotype consortium (IMPC) data

Dominic Norris (UK), Ruairidh King (UK) and Daniel Delbarre (UK) led this workshop introducing the IMPC, which is generating broad spectrum multi-dimensional phenotyping data for null alleles of every protein-coding gene in the mouse. This provides novel candidate genes for any given disease and provides phenotypic data for characterised and uncharacterised genes. The workshop highlighted the PCD-relevant phenotyping tests available and included an interactive session on how to access freely available data and navigate the IMPC portal. This provided delegates with the necessary skills to search for information on the genes or phenotypes of interest which can then be utilised to inform future studies.

#### Nasal NO in the diagnostic workup of PCD: clinical decisions and hands-on workshop

Jane Lucas (UK), Claudia Kuehni (Switzerland), Amanda Harris (UK) and Bruna Rubbo (UK) discussed the recommendations from the ERS guidelines for diagnosis of PCD [51, 76], which include that nNO should be used in the diagnostic work-up of patients 6 years and older using a chemiluminescence analyser with velum closure; those under 6 years should attempt nNO with tidal breathing; and that if there is a strong clinical history to support diagnosis, further investigations should be pursued even if nNO is normal. The workshop concluded with an interactive session learning to use the NIOX VERO machine.

#### Imaging modalities in PCD – recent research advances and the role of new techniques in clinical practice

Tom Semple (UK) began by demonstrating the appearances of abnormal *situs* (*inversus* and heterotaxy syndromes) on x-ray and CT, followed by an interactive session examining various clinical cases. Tom then discussed structural magnetic resonance imaging (MRI) and outlined the fundamental strengths and weaknesses of CT vs MRI. He discussed methods for deriving quantitative measures of disease severity from each modality and their limitations. The use of visual scoring systems in current clinical imaging practice has disadvantages, including the significant inter-reader variability, time required for scoring and specialist training. New respiratory applications of MRI and CT were discussed including quantitative CT and ventilation-perfusion MRI with preliminary data from Fourier decomposition ventilation-perfusion MRI, oxygen enhanced MRI and the application of deep learning imaging research.

#### Immunofluorescence microscopy as a diagnostic tool for PCD

The workshop started with a brief introduction about what an antibody is, types of hosts and differences between monoclonal and polyclonal antibodies. Niki Tomas Loges (Germany) then explained the basis of immunofluorescence. The University of Münster immunofluorescence protocol was reviewed as an example, including fixation; permeabilisation; blocking; incubation with the primary antibody and incubation with the secondary antibody. Niki also showed examples of different antibodies that are available to detect defects of ODA- and IDA- complexes, the radial spoke, the Nexin-Dynein regulatory complex and microtubule disorganisation defects. The workshop discussed trouble shooting for common technical difficulties and quality control of primary and secondary antibodies.

#### Physiotherapy – airways clearance –quality as well as quantity!

Gemma Marsh (UK), Lynne Schofield (UK) and Hannah Wilkins (UK) led an interactive problem-based session which examined the various physiotherapy techniques to manage the upper and lower airways in PCD [23, 90]. This involved a presentation by a local patient who shared his experience of daily physiotherapy, demonstrated his routine and explained its impact on his life. The roles of exercise, posture and continence were explored. The delegates split into smaller breakout groups to discuss specific cases and this also provided an interactive platform to share and explore their own challenging cases.

#### How do the cilia in a cell co-ordinate their motion – what we know and what is left to understand

This workshop, facilitated by Pietro Cicuta (UK) and Dominic Norris (UK), addressed how cilia are coordinated, both from experimental and conceptual methodological approaches. This was achieved using a ‘question and answer’ session. Pietro discussed the air-liquid interface (ALI) epithelial cell model he currently utilises to assess the co-ordination of ciliary beating [91]. This method enables longitudinal visualisation of the cells as they proliferate and expand on the transwell membranes. When flow is added to growing cells at the ALI, the cilia align; even when flow is subsequently removed the cilia on these cells remain aligned. However, flow does not cause cilia to align if applied only once the cells are fully differentiated, indicating that there is a window of time when this is possible. Adding artificial flow provides a modality for representing respiratory flow in vitro. There was active discussion and interaction during the session. However, the question of what enables the ‘healthy’ coordination of cilia in tissues is still far from understood.

#### Applying for ERS fellowships: how to increase your chances

Claudia Kuehni (Switzerland) and Myrofora Goutaki (Switzerland) summarised a wide range of ERS fellowship opportunities, including practical information such as the person specification for each fellowship and the timelines for application [92]. Opportunities are: the short and long-term research fellowships (ranging from 3 to 12 months in duration), the Marie Curie/Respire − 36 months long), clinical training fellowships to develop specific skills, guideline methodology with Cochrane based in Spain or NICE based in the UK and also public health fellowships which give practical experience on population-based research. Claudia and Myrofora gave feedback based on personal experience from the perspective of both the evaluator and applicant. This was followed by an interactive question and answer session.

#### How to establish and develop a PCD diagnostic facility

Claire Hogg (UK), Jane Lucas (UK), Andreia Pinto (Portugal) and Nisreen Rumman (Palestine) started the session by providing an overview of the PCD diagnostic tests including the standards discussed in the ERS diagnostic guideline [51, 76, 93]. They also shared their own experiences of setting up and maintaining a diagnostic service in different environments – including examples of how the EU PCD Consortia of BEAT-PCD, BESTCILIA and the PCD Taskforces have allowed the collaboration, mentorship and support necessary to develop the required skill sets across Europe and other nations around the world. They discussed the minimum requirements in PCD testing to secure a diagnosis and how to access training and support to develop specific services, with particular reference to contacting experts through the PCD Reference Centre Network established with the ERN-Lung programme. Nisreen Rumman discussed the approaches and challenges of setting up a new diagnostic service in Palestine [81] and Andreia Pinto presented her experiences from Portugal.

## Consensus statement workgroups

### Developing a validation study for the definition of pulmonary exacerbations

This workshop led by Claudia Kuehni (Switzerland) and Siobhán Carr (UK) was attended by delegates from varied clinical backgrounds. The aims were to review the development of the 2018 Expert Definition of Pulmonary Exacerbations in Clinical Trials Consensus and to explore the options for a future validation study and discuss different approaches [10].

It was discussed that the optimal approach would be a prospective multi-centre study capturing detailed standardised information on all items used to define an exacerbation, such as patient reported symptoms, measured traits (such as temperature) and investigations. Ideally it should include not only the items that were finally included in the definition, but also those that were discussed but discarded. In the interim, the first step agreed was to analyse the datasets recorded in the Azithromycin RCT [49] and PROVALF-PCD [44]. Most items used for defining an exacerbation had been assessed in these studies, although not exactly in the same manner. It was also agreed that the items used to define an exacerbation should be used in all clinical studies on PCD in the future.

### International consensus group for immunofluorescence microscopy to diagnose PCD

Heymut Omran (Germany) chaired a discussion on the need to establish a consensus guideline for the use and reporting of immunofluorescence in diagnostic PCD testing. Discussions focussed on practical techniques which are largely protocol driven and vary between centres (including sample collection, solutions for suspensions, storage methods and slide types). Particular focus was given to the importance of quality control. The potential to collaboratively compare test results between laboratories using a ring trial was discussed. Developing standards for immunofluorescence reporting was also discussed, including comments regarding the quality of samples. An email list was generated and circulated amongst attendees with the intention of establishing an immunofluorescence network.

## Poster session

The BEAT-PCD Conference and Training school included a poster session. Titles and authors are summarised in Table 1.

### Case Presentations

#### Difficult diagnostic cases

The diagnosis of PCD remains complex, even with the publication of evidence-based guidelines as there are still many subtle cases were investigations remain inconclusive [1, 76, 94]. For this reason, the BEAT-PCD Conference and Training School included an open forum to review difficult diagnostic cases across centres.

Suzanne Crowley (Norway) presented a term infant with neonatal pneumonia and persistent wet cough, especially related to feeds. He was initially thought to have unsafe swallow with possible aspiration but later showed more typical features of PCD with upper and lower airway disease. Investigations were unusual, revealing a nNO of 50 ppb, sparse and short cilia on several brushings and it was unclear if these were primary or secondary abnormalities. Genetic testing was negative for genes associated with reduced generation of motile cilia (including *CCNO* and *MCIDAS* [95]) but showed two variants in *DNAH9*. Defects in this gene were reported to cause absent ODA in the distal part of the cilium [96]. The patient’s immunofluorescence showed that DNAH9 was not absent but mislocalised and that there was a lack of cilia. Further diagnostic steps were discussed, including ALI culture to assess ciliogenesis and testing the *DNAH9* variants at the RNA level in the nasal brushing, or even whole exome/genome sequencing to look for another causative gene.

Sandra Rovira (Spain) presented a 48-year-old patient with recurrent upper airway symptoms, SIT, chronic sinusitis and recurrent suppurative OM since childhood. There was no significant lower airway disease. Investigations for cystic fibrosis, immunodeficiency and allergies were negative. PCD diagnostics showed normal nNO (986 ppb), normal CBF but areas of stiff dyskinetic cilia, and pathogenic genetic variants. Interestingly, immunofluorescence showed unusual staining for *DNAH5* with more intense fluorescence in the proximal axoneme. Assembled experts commented that these appearances have been observed by others but that they normalised upon repeat testing or ALI culture. There was agreement about the value of genetic testing in difficult diagnostic cases.

Lynne Schofield (UK) presented a Pakistani family with 3 siblings who had wet cough, chest infections, rhinorrhoea and hearing deficits, without laterality defects or bronchiectasis. Their mother also had wet cough with rhinorrhoea. Nasal NO was 160 ppb and 3 ppb for the two siblings who were able to perform the test. Other PCD diagnostics showed heavy mucous impedance, dyskinetic cilia with a staggered or double beat and with tips bent at 90 degrees, but CBF and TEM were normal. Immunofluorescence and ALI cultures were unsuccessful, and genetic testing varied between individuals within the family. In discussion it was raised whether this family truly has PCD or another muco-ciliary clearance abnormality. The group agreed that PCD diagnosis cannot be confirmed without TEM or genetics, but patients should be managed as PCD until proven otherwise. It was also discussed that the characteristic double beat which has been seen in other cases of PCD, is a strong indicator that further testing is required to confirm or exclude a diagnosis.

Laura Gardner (UK) presented a 6-month-old boy with levocardia, abdominal SI, left atrial isomerism and functional hyposplenia. He had no respiratory symptoms but developed neonatal seizures which were likely secondary to cerebral infection. This warranted genetic testing through the Next Generation Children Project [97], which revealed 2 mutations in *DNAH11* (one pathogenic and another missense variant of unknown significance). Despite an absence of symptoms, PCD diagnostics were pursued and showed subtle stiff cilia on HSVA but normal TEM and immunofluorescence. Given that around 30% of patients with PCD have apparently normal ciliary ultrastructure on TEM [98], including associated with *DNAH11* mutations, 3D electron tomography was pursued. Findings were consistent with a *DNAH11* genetic defect, with an ODA to microtubular doublet volume ratio of 10.24%. This is in keeping with published data showing a median ODA to microtubular doublet volume of 10.3% (interquartile range 9.3–10.5%) in *DNAH11* subjects, compared to 13.8% (12.9–14.4%) in controls [98]. It is therefore likely that *DNAH11* c10472G > A; p.(Arg3491His) is a novel pathogenic mutation associated with PCD. However, it was questioned whether this patient can be labelled as PCD given his relative lack of respiratory symptoms. The group agreed that clinical follow-up is warranted as PCD has a spectrum of clinical severity across known structural and genetic mutations.

Randy Suryadinata (Australia) presented a 2.5-year-old boy with chronic cough, recurrent otitis media and persistent nasal discharge. There was no neonatal respiratory distress or laterality defects. Genetic testing revealed two heterozygous mutations in *HYDIN* in cis (which were shared with the unaffected mother and sister), and an additional mutation in *DNAH11*. HSVA showed reduced beat amplitude and circling movements. TEM was unremarkable, but tomography exposed a possible volume loss of the *HYDIN* projection, described as a ‘missing bridge’. The questions raised with this case were whether a digenic inheritance pattern involving 2 combined mutations (*HYDIN* and *DNAH11*) can cause PCD, or whether the specific *HYDIN* mutations could cause a dominant form of PCD. It was noted that *HYDIN* mutations can be difficult to define, due to the *HYDIN2* copy gene, as well as it being large gene with multiple polymorphisms. It was thought that a dominant inheritance was unlikely. Segregation studies, testing the mother and sister who share the mutations, were suggested. Linkage studies by haplotype testing may also be informative.

Nagehan Emiralioglu (Turkey) presented two cases. A 3-year-old boy with failure to thrive, developmental delay, polyuria and polydipsia. He was born at term to consanguineous parents without neonatal complications. Investigations revealed features of Fanconi Bickel syndrome which was confirmed by genetic testing (*SLC2A2* gene). At age 4, he presented to respiratory services with chest infections, persistent wet cough, recurrent rhinosinusitis, and SIT. HSVA on cell culture revealed dyskinetic cilia, TEM is still pending. Genetic experts at the meeting discussed potential for a modifier gene effect, especially since the gene for Fanconi Bickel syndrome maps to chromosome 3q26.1-q26.3, and *CCDC39* to chromosome 3q26.33.13. The group commented that “contiguous gene deletion syndrome” can be another possible reason for this, as described with *DNAH5* and cri du chat syndrome [99]. It is therefore warranted in this case to check if *CCDC39* is absent by immunofluorescence, and also perform karyotyping to check for deletions. In the second case, a 4-month-old infant, born at term to consanguineous parents with neonatal respiratory distress, SIT and skeletal dysplasia, to consanguineous parents. He presented to respiratory services with recurrent wheezing, pneumonia and persistent nasal discharge. His mother has bronchiectasis and history of lobectomy, two uncles and grandfather also have bronchiectasis. HSVA for both the infant and mother revealed almost static cilia. The question was raised about the genetic causes of skeletal dysplasia as part of a primary ciliopathy with a respiratory phenotype. The group commented that there is increasing reports of primary ciliopathies having motile ciliary defects as well [100]. It was noted that in fish models if you knock out any PCD gene, the fish develops scoliosis [101]. Some have observed scoliosis in PCD patients but whether there is a link between remains unknown.

#### Difficult management cases

The limited evidence-base for best clinical practice in PCD means that management can be challenging for individual clinicians, even in larger centres [1, 102]. BEAT-PCD included an interactive session to discuss difficult cases with the hope of consolidating collective experiences and developing an expert consensus.

Vendula Martinu (Czech Republic) presented the case of a 13-year-old girl with PCD (*DNAH5*, ODA defect), recurrent pulmonary exacerbations and faltering growth. Bronchoalveolar lavage (BAL) revealed *Exophiala dermatidis*. The patient commenced on voriconazole but unfortunately developed long QT-syndrome. *Exophiala* growth persisted with evidence of voriconazole resistance. Chest CT showed extensive bronchiectasis and air trapping. There was no sign of allergic sensitisation to fungus and the patient had normal immune function. The discussion focussed on dual -azole therapy, drug monitoring and inhaled antifungal treatments.

Marco Poeta (Italy) presented four cases of persistent wet cough with diverse diagnoses highlighting the importance of differential diagnosis and comprehensive investigation. The first two children were diagnosed with congenital vascular anomalies. One patient underwent aortopexy with resolution of her symptoms. The third case was diagnosed with type 2 congenital pulmonary airway malformation and symptoms resolved following lobectomy. The fourth case was a 7-year-old with recurrent lower respiratory tract infections, decreased lung function and *Pseudomonas aeruginosa* in her sputum. PCD diagnostics showed nNO 7 ppb, ODA defect on TEM and genetics are awaited.

Anne Schmidt (UK) presented the case of a 19-year-old woman with bronchiectasis, hydrocephalus and bilateral hydrosalpinx. She presented at 6 weeks old with aqueduct stenosis and hydrocephalus requiring insertion of a ventriculoperitoneal shunt. She underwent diagnostic work-up including PCD diagnostics at age 6 and 14 years. Results showed normal nNO and TEM but a low ciliary beat frequency (CBF) and a stiff dyskinetic beat pattern increasing the suspicion of a ciliopathy. At 15 years, she developed a significant drop in lung function (FEV_1_ 80 to 54%) with *Aspergillus fumigatus* infection on BAL. She was managed with intravenous antifungals but continues to have recurrent chest infections caused by other bacterial organisms. This case stimulated discussion on whether this represented fungal bronchitis and various treatment strategies were shared by clinicians with similar patients.

## Early stage researchers networking forum

The BEAT-PCD Training School hosted the 2nd Early Stage Researchers (ESR) networking forum led by ESR representatives Bruna Rubbo (UK) and Myrofora Goutaki (Switzerland). The networking forum brought together 39 delegates, including postgraduate students, clinicians and postdoctoral researchers. Feedback from the inaugural ESR forum held in the previous Conference [19] requested the formation of small break-out groups to discuss hot topics in PCD research. Participants proposed various subjects, from animal models to developing a grant proposal. Each break-out group discussed their topic and elected a speaker to summarise the important points to the larger collective. Contact details were exchanged amongst participants with a plan to share ideas and expertise on future collaborative projects.

## Evaluation feedback from participants

**Fig. 2:**
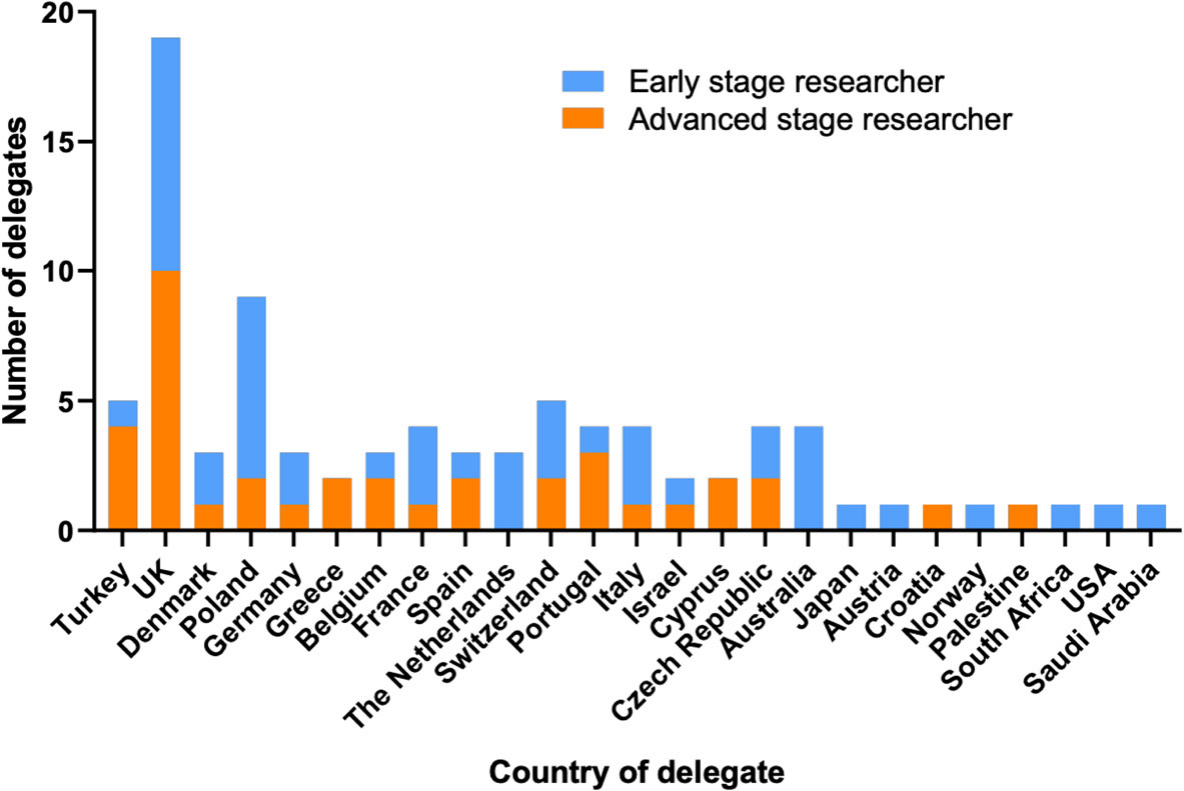
Country of work of respondents of the feedback survey for the 4th BEAT PCD Conference and 5th PCD Training School. ESR: early stage researcher, ASR: advanced stage researcher

An online feedback survey was circulated to the 143 delegates following the Conference and Training School. Eighty-eight respondents (62% response rate) from 25 countries completed the survey (Fig. 2) with an average rating of 9.1/10 for the conference and 8/10 for workshops.

## Summary

BEAT-PCD can be characterised as a successful COST-ACTION, not only through the successful completion and publication of multiple studies which have advanced our understanding of PCD, but also through its contribution towards the training of young researchers, facilitation of efficient networking which has led to the development of collaborative projects and the dissemination of expertise across the world. Moreover, almost all the participants of BEAT-PCD were involved in multiple projects, from STSMs to manuscript preparation. This indicates how interactive the COST-action has been and the genuine enthusiasm and excitement it has invoked amongst its participants, as also shown by the high response numbers and positive comments received through the end of meeting feedback survey and the uptake for participation in the future PreMedCilia COST application. BEAT-PCD has built upon the previous work undertaken by BEST-CILIA and has set-up a high profile and important platform for an exciting, productive and necessary continuation through follow-on funding streams.

## Data Availability

For data and materials relating to this document please contact the corresponding author: Prof Claire Hogg c.hogg@rbht.nhs.uk

## Abbreviations

ALI: Air liquid interface
ASR: Advanced stage researcher
BEAT-PCD: Better experimental approaches to treat PCD
CBF: Ciliary beat frequency
CT: Computed tomography
ENT: Ear, nose and throat
ESR: Early stage researcher
ET: Electron tomography
FEV_1_: Forced expiratory volume in 1 s
HSVA: High speed video-microscopy analysis
IDA: Inner dynein arm
iPCD: international PCD (Registry)
MDT: Multidisciplinary team
MRI: Magnetic resonance imaging
N-DRC: Nexin-dynein regulatory complex
nNO: nasal nitric oxide
ODA: Outer dynein arm
PCD: Primary ciliary dyskinesia
PICADAR: PrImary CiliARy DyskinesiA Rule
QOL-PCD: Quality of life primary ciliary dyskinesia
RCT: Randomised controlled trial
SA: *Situs ambiguous*
SI(T): *Situs inversus* (*totalis*)
SRM: Super-resolution microscopy
STSM: Short term scientific mission
SS: *Situs solitus*
TEM: Transmission electron microscopy
